# Down Syndrome, Increased Risk of Dementia and Lipid Disturbances

**DOI:** 10.34763/devperiodmed.20172101.6973

**Published:** 2017-05-29

**Authors:** Anna Kłosowska, Agnieszka Ćwiklińska, Agnieszka Kuchta, Agata Berlińska, Maciej Jankowski, Jolanta Wierzba

**Affiliations:** 1 Department of Pediatrics, Hematology and Oncology Gdansk Poland; 2 Department of Clinical Chemistry Gdansk Poland; 3 Department of General Nursery Medical University of Gdansk, Gdansk Poland

**Keywords:** Down syndrome, Alzheimer disease, dementia, lipids, zespół Downa, choroba Alzheimera, otępienie, lipidy

## Abstract

Down syndrome (DS) is the most common chromosomal aberration and genetically determined cause of intellectual disability. DS patients often present with some congenital defects and chronic diseases, including early onset dementia, which affects 70% of DS patients over 55 years of age and has a clinical presentation similar to Alzheimer disease (AD). The symptoms of DS originate from excessive genetic material within the “critical region” of the 21^st^ chromosome. The “critical region” encompasses genes potentially associated with increase risk of dementia, e.g. the APP gene (amyloid beta precursor protein) which leads to excessive amyloid beta production. Post-mortem studies of DS patients’ brains revealed diffuse deposition of the insoluble form of amyloid beta (Aβ), which is a characteristic feature of AD. Moreover, those changes were commonly observed in subjects > 31 years old. The pathomechanisms of AD have not been fully elucidated and scientists are still searching for new risk factors that may contribute to the development of this common illness. Recent research proved that lipid disturbance, especially disorders in the metabolism of HDL (high density lipoprotein) may play a crucial role in this pathogenic process. There are many studies examining lipid and lipoprotein concentration in the DS population, but up to now there are insufficient studies comparing these parameters with memory impairment, which may be a useful model for better understanding of the dementia pathomechanism.

## Streszczenie

Zespół Downa jest najczęstszą aberracją chromosomową oraz najczęstszą przyczyną niepełnosprawności intelektualnej uwarunkowanej genetycznie. U pacjentów z zespołem Downa częściej występują wady wrodzone oraz choroby przewlekłe, takie jak wczesne otępienie, które dotyczy około 70% pacjentów powyżej 55 roku życia, a przebiegiem klinicznym przypomina chorobę Alzheimera (AD). Objawy charakterystyczne dla zespołu Downa są spowodowane nadmiarem materiału genetycznego zawartego w tzw. „regionie krytycznym” 21. chromosomu. Ten „region krytyczny” zawiera geny związane z rozwojem otępienia, między innymi gen APP (ang. Amyloid beta precursor protein) kodujący białko prekursorowe dla amyloidu beta (Aβ), którego nadmiar może skutkować zwiększoną produkcją Aβ. Badania pośmiertne mózgów pacjentów z zespołem Downa wykazały obecność rozlanych złogów nierozpuszczalnej formy Aβ, co jest jedną z charakterystycznych cech AD. Ponadto zmiany te występowały powszechnie u pacjentów powyżej 31 roku życia. Patomechanizmy rozwoju AD nie zostały do końca poznane i naukowcy nieustannie poszukują nowych czynników ryzyka, które mogą wpłynąć na rozwój tej częstej choroby. Ostatnie badania pokazują że zaburzenia gospodarki lipidowej, w szczególności dotyczące HDL (ang. High-density lipoprotein) mogą odgrywać znaczącą rolę w patogenezie AD. W piśmiennictwie dostępnych jest wiele prac dotyczących poziomu lipidów i lipoprotein w surowicy u pacjentów z zespołem Downa, ale brakuje badań opartych na wiarygodnych testach, porównujących te parametry z zaburzeniami pamięci. Takie badania naukowe mogłyby pomóc w zrozumieniu rozwoju otępienia i posłużyć jako model do dalszych badań nad AD.

## Introduction

Down syndrome (DS) is the most common chromosomal aberration and genetically determined cause of intellectual disability (ID). The occurrence of DS ranges between 1:625-1:847 live births [1, 2]. Besides ID, weak muscle tone and characteristic dysmorphy, DS patients commonly present with some congenital defects, including cardiac (40-50%) and gastrointestinal (about 12%) malformations. Moreover, other diseases are more prevalent in DS patients as compared to the general population, such as obesity, hypothyroidism, type 1 and 2 diabetes, coeliac disease, leukaemia and dementia [3]. Despite the increased risk of chronic disease, life expectancy of DS patients is continuously increasing. This can be explained not only by the growing number of successful pregnancies and deliveries by women aged >36, but mainly by the surgical correction of cardiac defects and the possibility of treatment of chronic conditions [4, 5]. In developed countries up to 96% DS children survive the 1^st^ year of life if they do not suffer from congenital heart defects. The rate drops to 80% when heart defects are present [6].

Currently, the mean life expectancy of DS patients reaches 55-60 years [6]. Because of this prolonged life expectancy, the overall medical profiles of DS patients have changed, as well as their healthcare needs. Available research results emphasize the significance to understand the development of early-onset dementia as well as obesity and metabolic disturbances in DS subjects, which can simultaneously lead to cerebral destruction and cerebrovascular problems and have a great impact on the quality of life among DS subjects and their families [7, 10].

## Ds syndrome and increase risk of dementia

More than 70% of DS patients surviving up to the age of 55-60 demonstrate early-onset dementia, which clinically presents with similar features to Alzheimer’s disease (AD-like disease)[3].

AD is a progressive neurodegenerative disorder with more than 30 million individuals affected worldwide. It is estimated that in the U.S. 12% of the people aged >65 will develop AD, with the percentage rising to 45% for individuals aged >80. Early signs of the disease consist of loss of short-term memory, learning ability, and linguistic fluency. Later on, disorders of long-term, semantic, and procedural memory follow. Finally, patients become fully dependent, losing basic skills such as the ability to move or eat. Patients diagnosed over the age of 65 are classified as late-onset (LOAD – late-onset AD), and those diagnosed under the age of 65 – early-onset (EOAD – early-onset AD). Among EOAD patients, a small group suffers from a familial form of the disease (EOFAD – early-onset familial AD) which is associated with amyloid beta precursor (APP) gene mutation or other presenilin genes. EOFAD cases represent only 5% of all AD patients, however studying the EOFAD defective genes has provided further insight into AD pathology [11].

The clinical presentation of DS originates from excessive genetic material carried by an additional full 21 chromosome or its partial trisomy. Research focusing on the “critical region” genes has helped to understand how excess of certain proteins affects the phenotypical features in DS and has been crucial to establishing genotype-phenotype correlation.

!e “critical region” encompasses more than 630 different genes, among them genes potentially associated with dementia development:

– *APP* (*amyloid beta precursor)* which, if overly expressed, leads to excessive amyloid beta (Aβ) production;
– *SOD1 (Superoxide dismutase1*) gene encoding superoxide dismutase which in excess destabilizes oxidoreductive balance and leads to premature aging, as well as immune disturbances;
– *CBS* (*Cystatione beta-synthetase*) gene associated with homocysteine metabolism;
– *DYRK1A* (*Dual specificity tyrosine-phosphorylation-regulated kinase*) gene coding the enzyme DYRK1A catalyzing autophosphorylation on serine-threonine and tyrosine residues, playing a role in multiple signaling pathways active during early development of the central nervous system. This enzyme’s abnormal activity is considered essential in DS brain development;
– *SYN1J* (*synaptojanin 1*) gene encoding an enzyme important in synaptic transmission [12].

One of the characteristic features of AD is the deposition of the so-called senile plaques in the brain, which include the insoluble form of Aβ. In a study of 29 DS patients aged 3-72 years amyloid deposits were already discovered in a 12 year-old child, and appeared universally in participants >31 years old. Post-mortem studies of DS brains reveal diffuse Aβ42 (a beta ending at amino acid 42) deposits and progressing to compacted, fibrillar plaques often containing Aβ40 (a beta ending at amino acid 40) that was associated with neurodegeneration [13]. The distribution of fibrillary plaques and neurofibrillary tangles in DS brains is very similar to AD, although the density is greater in DS [14]. Henceforth, DS patients seem to be a certain “clinical model” for AD research.

## Other risk factors for ad development

The pathomechanisms of AD have not been fully elucidated. It has been found that it is linked to both genetic and environmental factors, such as the lack of physical activity, cerebral hipoperfusion, abnormal glucose metabolism, oxidation-reduction imbalance or inflammatory processes [3, 11]. Risk factors for LOAD that have been known for a while include old age, a family history of dementia, and possession of one or more APOE ε4 alleles [15].

Recently, scientists proved that lipid disturbances can also be connected with the development of AD. Inter alia, an inverse correlation between the levels of Aβ and the concentration of high density lipoprotein (HDL) cholesterol in plasma has been observed[16, 17].

HDL is one of the major carriers of cholesterol in blood. Through its key role in reverse cholesterol transport, anti-inflammatory, antiaggregatory and antioxidant activities, it influences cardiovascular health in a positive way. Numerous epidemiological studies have shown that lifestyle interventions proven to increase HDL levels also provide neuroprotective effects [18, 19]. Moreover, elevated HDL levels are also associated with longevity, improved cognition and dementia–free survival [20]. HDL is the smallest and densest amongst lipoproteins. It is composed of an outer layer containing free cholesterol, phospholipids, and various apolipoproteins (Apo), and a hydrophobic core composed of triglycerides and cholesterol esters. HDL is particularly rich in Apo A-I and Apo A-II apolipoproteins. Apo A-IV, Apo C, and Apo E are also present [21]. Human cerebrospinal fluid (CSF) contains spherical lipoproteins resembling HDL in human plasma. Apo E and A-I are the major human CSF apolipoproteins. Astrocytes synthetize ApoE-rich HDL locally, while Apo A-I-rich HDL are derived from plasma HDL crossing the blood-brain barrier [22].

The relationship between HDL level, Aβ and brain function seems to be significant. Aβ production depends on cholesterol content in cellular membranes. HDL is able to suppress amyloid production by decreasing cellular cholesterol via activation of reverse cholesterol transport mediated by ABC transporters [23]. It is also known that HDL carries Aβ in CSF and plasma, and can directly bind the excess amyloid peptide which results in the inhibition of its assembly into fibrils [24]. Additionally, HDL can remove Aβ accumulated in vascular walls in the course of vascular dementia development, as well as reduce oxidative stress and thus indirectly reduce amyloid production [25]. The monomeric form of Aβ is a strong chelator of pro-oxidant metal ions and its production is enhanced by oxidative stress.

The relationship between HDL concentration and memory was investigated by the Whitehall II Study. A group of 3673 middle-aged adults was examined, and the results suggested a clear association between low plasma level of HDL and poor memory. Furthermore, decreased HDL was associated with decline in memory function over a 5-year long follow-up. The patients’ education, occupation, chronic diseases, or medication use did not change the results, and neither did the APOE e4 status. Serum levels of total cholesterol and triglycerides presented no link to memory deficit or decline. The study identified HDL molecules as important for memory, and low HDL in the middle-aged population as an important risk factor for dementia development later on [26]. However, some researchers studying the elderly population connected low HDL concentration with vascular dementia but not with AD [27]. Such equivocal findings may be explained by dementia itself modifying lipid levels [28]. In the future, it might be necessary to assay lipid concentrations before diagnosing dementia.

Other studies have shown that atherosclerosis and atherosclerosis – related diseases, such as coronary artery disease (CAD), or type II diabetes mellitus are also associated with LOAD[29]–[31]. In a 1990 study, abundant senile plaques were found in the brains of non-demented patients dying with or as a result of critical CAD (75%) compared to non-heart disease subjects (12%)[29]. It is now recognized that individuals with heart diseases often have demonstrable AD-like Aβ deposits within neurons in the brain and that cerebral atherosclerosis is strongly associated with an increase frequency of neurotic plaques. [32].

## How does this relate to the ad – Like disease in ds?

In the past, individuals suffering from DS were considered protected from atherosclerotic disease, one of the factors predisposing to AD. Murdoch described a complete lack of atherosclerosis in five institutionalized DS patients, and classified DS as an “atheroma-free” disease model [33]. A series of subsequent post mortem studies demonstrated lower atheroma incidence in institutionalized DS patients as compared to controls of the same age [7, 34]. Nevertheless, there are also opposing reports not supporting DS as an “atheroma-free” disease model. Autopsy material coming from institutionalized children and adolescents <21 years of age with intellectual disability reveals slightly more advanced atherosclerosis of coronary arteries and aortas as compared to other study subjects [8]. Recent data collected by two statistically significant epidemiological studies targeting DS patients suggests an increased mortality risk due to ischemic heart disease and cerebrovascular disease in comparison with the general population. Scientists from Sweden and Denmark noted that individuals suffering from DS have a 3.9-fold higher predisposition to dying from ischemic heart disease [35]. Another epidemiological study of >14 000 individuals from California found a similar 4.3-fold increase of mortality due to ischemic heart disease [36]. As it was mentioned before, coronary artery disease remains one of the risk factors for AD.

Many research projects focused on comparing lipid and lipoprotein concentrations in DS and non-DS groups, however, produced equivocal conclusions [9, 37, 38, 39, 40, 41]. The aforementioned studies varied significantly in their size, specific outcomes, and control groups. Only two of them discuss the association of DS with an unfavorable lipid profile independent of the individuals’ body mass. One cross-sectional study observed prepubertal participants (aged 4-10) with BMI below the 95^th^ percentile for their age and sex focusing on lipid profiles, and compared the results with those of their non-DS siblings. Children with DS had higher total cholesterol, LDL, TG, and lower HDL than their siblings, the outcomes did not vary depending on body mass [40]. This is in agreement with our own initial results.We have compared serum lipid profiles, total cholesterol, LDL, TG and HDL level in 34 children with DS and 31 non-DS children aged from 8 to 18 years. Children with DS had higher TC (difference 15 mg/dl), LDL (19.9 mg/dl), TG (23.6 mg/ dl) after adjustment for age, gender and BMI. We did not notice a significant difference in HDL levels in this group. Another study targeted DS fetuses at 18-36 weeks of gestation. Gestational age, size, and number of subjects were taken into account. DS fetuses exhibited significantly higher cholesterol levels [41]. Congenital heart disease, hypothyroidism, body mass, glucose levels, and insulin levels alone are unlikely to explain the difference between healthy and DS children’s lipid profiles. The possible influence of chromosome 21 overexpression influencing the lipid profile remains a question yet to be answered.

No studies linking lipid concentrations and memory in DS patients are currently available. As mentioned before, Aβ deposition in DS patients’ central nervous system was seen as early as at the age of 12, and became universal by the age of 31. It is important to develop studies focusing on children and young adults with DS, because their results might appear beneficial in understanding the correlation between lipid metabolism and dementia.
